# Gold-Deposited Nickel Foam as Recyclable Plasmonic Sensor for Therapeutic Drug Monitoring in Blood by Surface-Enhanced Raman Spectroscopy

**DOI:** 10.3390/nano10091756

**Published:** 2020-09-06

**Authors:** Saiqa Muneer, Daniel K. Sarfo, Godwin A. Ayoko, Nazrul Islam, Emad L. Izake

**Affiliations:** 1 School of Chemistry and Physics, Science and Engineering Faculty, Queensland University of Technology, 2 George St., Brisbane QLD 4000, Australia; saiqa.muneer@hdr.qut.edu.au (S.M.); daniel.sarfo@qut.edu.au (D.K.S.); g.ayoko@qut.edu.au (G.A.A.); 2 School of Clinical Sciences, Faculty of Health, Queensland University of Technology, 2 George St., Brisbane, QLD 4000, Australia; nazrul.islam@qut.edu.au

**Keywords:** meropenem, nickel foam, electrodeposition, therapeutic drug monitoring, surface enhanced Raman spectroscopy, HPLC-SERS, recycled gold nanosensor

## Abstract

A sensitive and recyclable plasmonic nickel foam sensor has been developed for surface-enhanced Raman spectroscopy (SERS). A simple electrochemical method was used to deposit flower-shaped gold nanostructures onto nickel foam substrate. The high packing of the gold nanoflowers onto the nickel foam led to a high enhancement factor (EF) of 1.6 × 10^11^. The new SERS sensor was utilized for the direct determination of the broad-spectrum β-lactam carbapenem antibiotic meropenem in human blood plasma down to one pM. The sensor was also used in High Performance Liquid Chromatography (HPLC)-SERS assembly to provide fingerprint identification of meropenem in human blood plasma. Moreover, the SERS measurements were reproducible in aqueous solution and human blood plasma (RSD = 5.5%) and (RSD = 2.86%), respectively at 200 µg/mL (*n* = 3), and successfully recycled using a simple method, and hence, used for the repeated determination of the drug by SERS. Therefore, the new sensor has a strong potential to be applied for the therapeutic drug monitoring of meropenem at points of care and intensive care units.

## 1. Introduction

Sensitive detection of biomolecules, particularly drugs, are of vital importance in sample analysis and process monitoring. The chromatographic methods like high-performance liquid chromatography (HPLC-MS) and gas chromatography (GC-MS) have been used previously for the determination of drugs in biological fluids [1]. Mass spectroscopy is considered as one of the gold standard methods due to molecular structure identification and accurate determination of drugs in blood [2]. However, these techniques are laborious, require expensive equipment, long incubation times, and highly trained personal. Also, they are not cost-effective or time saving in high throughput screening of many samples. Many researchers coupled vibrational spectroscopy with chromatographic techniques; however, this coupling of HPLC-based separation and vibrational spectroscopy has not been significant over the years, resulting in further complexity produced by using HPLC solvents, which often mask the analyte signals detected by vibrational spectroscopy. To reduce the subsequent false identification, there is a need to use a sensitive and robust technique coupled with HPLC [3,4,5]. Out of other vibrational spectroscopies, surface enhanced Raman spectroscopy (SERS) has been demonstrated in recent years for the therapeutic drug monitoring (TDM) of various drugs as well as their metabolites in biological samples [6]. SERS has many advantages such as high sensitivity, non-destructive nature, capacity to provide molecular fingerprint identification of the analyte, the capacity to measure analytes in biological fluids [7,8]. Therefore, SERS would also be another gold standard analytical method for therapeutic drug monitoring in blood in addition to mass spectroscopy.

In SERS, the analyte molecules are adsorbed onto a nanostructured noble metal substrate. When incident light of optimum frequency interacts with the substrate, it excites the surface plasmons of the nanostructures as well as the Raman photons from the analyte [9]. Therefore, the weak Raman emission of the analyte experience strong amplification due to its coupling with the electromagnetic field of the excited surface plasmons (electromagnetic effect) [10]. Besides, the development of a charge-transfer complex between the analyte and the substrate facilitates the transfer of electrons between adsorbed molecules and the substrate in the ground and excited states of the analyte-metal system. This leads to additional enhancement of the Raman signal of the analyte (chemical effect) [11]. The SERS substrates can be either colloidal (3D) or a solid (2D) substrate. Colloids of noble metals (e.g., gold and silver), are frequently used as substrates as they provide strong signal enhancement. However, using colloidal substrates for SERS measurements does not provide reproducibility as it is difficult to control the aggregation of the nanoparticles and generate efficient hotspots. Also, the net charge of the analyte molecule can greatly influence its adsorption onto the metallic nanoparticles. [12,13]. To address these challenges, solid SERS substrates have been synthesized by a variety of methods. For example, highly ordered metallic substrates have been fabricated using lithographic, chemical ion etching, and picosecond/femtosecond laser ablation techniques to provide reproducible SERS measurements [11,14]. Other methods, such as direct laser writing and electrochemical deposition, have been also used to synthesize randomly patterned substrates for the detection of various analytes [15,16].

In this work, we demonstrate a plasmonic nickel foam nanomaterial as a recyclable and sensitive SERS substrate for the rapid detection of drugs in human blood plasma samples. Nickel foam has been reported as a SERS sensor in literature before [17]. The new substrate was fabricated by a simple and rapid electrochemical method where a forest of flower-shaped gold nanostructures was deposited onto the porous structure of the nickel foam. The new substrate was used to detect meropenem (MPN), a broad spectrum β-lactam antibiotic, in human blood plasma, for the first time, by SERS. The new sensor was also used as a detector in an HPLC-SERS arrangement for the quantitative analysis of MPN in human blood plasma.

The antibiotic MPN is used in intensive care units to treat bacterial meningitis, pneumonia, sepsis, febrile neutropenia, peritonitis, also gynecological, intra-abdominal, and other severe infections. MPN has low stability in aqueous solutions due to the hydrolysis of its β-lactam ring, especially under acidic or basic environment. The administration of a high dose of MPN to patients with renal dysfunction can lead to the accumulation of the drug, thus leading to undesired adverse effects. Therefore, it is important to monitor the stability of MPN injections and for its TDM in patients under treatment with large doses of the drug [18].

The determination of MPN is usually carried out by immunoassay and chromatographic methods [19,20]. The detection limit is 25 ng/mL (6.5 × 10^−8^ M) by HPLC [19] and by 2 ng/ mL (5.2 × 10^−9^ M) by ELISA [20]. The immunoassay methods, which are usually time-consuming are not readily adapted for the TDM of MPN due to its thermal instability above room temperature and shorter plasma half-life. Also, chromatographic methods usually require sample preparation steps and use of liquid solvents, which can lead to degradation of MPN during analysis, the use of expensive equipment (e.g., diode array or mass detectors), and trained personnel [1,21]. Therefore, the new SERS method for the screening of MPN offers a sensitive and rapid molecular structure identification of MPN in blood.

## 2. Materials and Methods

### 2.1. Materials

Nickel Foam (200 × 300 × 1.6 mm, average hole diameter = 0.25 mm) was purchased from MTI corporation Ltd. (Richmond, CA, USA). Pharmaceutical grade meropenem 10 g (SKU: 137-15674; CAS: 119478-56-7) was purchased from Novachem (Heidelberg, Australia). Ammonium acetate, plasma from human, gold chloride (HAuCl_4_), perchloric acid (HClO_4_), 2-Quinoline, thiol and acetonitrile were purchased from Sigma-Aldrich (St. Louis, MO, USA). The plasma from a human was sourced under human research ethics exemption granted by Queensland University of Technology (number 1800001209). All aqueous solutions were prepared using deionized water (18.2 MΩ cm).

### 2.2. Instrumentation

μAutolab potentiostat (Metrohm, Herisau, Switzerland) was used to deposit gold nanostructures onto nickel foam electrochemically. Scanning electron microscopy (SEM) measurements were carried out using the Tescan, MIRA 3 microscope (Tescan Orsay Holding, Brno, Czech Republic). A handheld Raman spectrometer (ID Raman mini 2.0, Ocean Optics, Dunedin, FL, USA; spectral resolution 12 cm^−1^) was used for the Raman measurements and operated in the raster orbital scanning mode (ROS). The measurements were carried out in the wavelength range of 400 cm^−1^ to 2000 cm^−1^. Sample excitation was carried out using a laser source of 785 nm and a laser power of 5 mW. The SERS spectra were collected using 10 accumulations and an acquisition time of 1 s per measurement. The background noise and fluorescence in the Raman measurements were automatically corrected by the instrument software algorithm (OceanView Spectroscopy 1.5.07, Dunedin, FL, USA) [16,22]. The high-performance liquid chromatography (HPLC) measurements were carried out using Agilent HPLC Series 1100 with a Diode array detector (Waldbronn, Germany) and the detector was set at 298 nm. The HPLC analysis was carried out using Value Solution ChemStation, version 4, by Agilent technologies, Waldbronn, Germany, 2010.

### 2.3. Pre-treatment of the Nickel Foam Substrate

The nickel foam was cut into a 5 mm × 12 mm rectangle shape and successively sonicated for 10 min in acetone, ethanol, and ultra-pure water. The foam was then dried under a stream of nitrogen gas before the electrochemical deposition of the gold nanostructures.

### 2.4. Synthesis of Plasmonic Nickel Foam by Chronoamperometry

Chronoamperometry was used to deposit gold nanostructures onto the nickel foam using a standard cell setup of three electrodes comprising nickel foam as the working electrode, a reference electrode of an Ag/AgCl (in saturated KCl) and platinum wire as the counter electrode. The deposition of the gold nanostructures electrochemically was conducted using 10 mL of 4 mM HAuCl_4_ solution and NaClO_4_ as a supporting electrolyte. The electrochemical deposition was carried out for 600, 900, and 1200 s at a negative potential of −0.08 V and an equilibration time of 5 s [21]. The substrates were then air-dried and subsequently put in an oxygen plasma oven for 10 min to remove the weakly adsorbed organic molecules that may contaminate the deposited gold nanostructures.

### 2.5. Characterization of the Plasmonic Nickel Foam Material

SEM was used to determine the shape and average size of the gold nanostructures on the nickel foam. Energy-dispersive X-ray spectroscopy (EDX) was used to confirm the chemical composition of the deposited nanostructures by using TESCAN MIRA 3 (Tescan Orsay Holding, Brno, Czech Republic). Nitrogen adsorbed BET surface area was performed after degassing the samples (100 °C, 24 h) using a Micromeritrics 3-flex 2020 (Norcross, GA, USA).

### 2.6. Calculation of the Enhancement Factor (EF) of Plasmonic Nickel Foam Material

2-Quinolinethiol (2-QT) was used as a Raman probe to determine the SERS enhancement factor (EF) of the plasmonic nickel foam [23]. Then, 100 µL aliquots of 10^−2^ M and 10^−12^ M of 2-QT were loaded onto bare and gold-nanostructured nickel foam substrates, of similar dimensions (5 mm × 12 mm), for 15 min. The substrates were then washed using distilled water, dried under a stream of nitrogen gas, and screened by SERS. The signal intensity of 2-QT at 1368 cm^−1^ was monitored and the EF was calculated using the equation, EF = I*_SERS_*/I*_R_* × C*_R_*/C*_SERS_* [24].

### 2.7. Preparation of Standard Solutions

For the SERS quantification, a 5 × 10^−4^ M (200 µg/mL) MPN stock solution was prepared by dissolving 1 mg of the drug in 5 mL of deionized water. Dilute solutions of MPN in the range of concentrations 10^−6^ M to 10^−12^ M were prepared by serial dilution with deionized water.

### 2.8. Determination of MPN by SERS

A hundred μL of the MPN in aqueous solutions in the concentration range of 200 μg/mL to 3.8 pg/mL (5 × 10^−4^ M to 10^−12^ M) were loaded onto plasmonic nickel foam substrates and allowed to stand for 15 min. The loaded substrates were washed using deionized water, air-dried, screened by SERS, and the signal intensity of MPN at 1546 cm^−1^ plotted against the log of MPN concentration. Another standard curve was plotted by loading MPN dissolved in human blood plasma in the same concentration range of 5 × 10^−4^ M to 10^−12^ M on individual nickel foam substrates [5].

### 2.9. Recycle of Plasmonic Nickel Foam

To recycle the used plasmonic nickel foam, it was dipped in absolute ethanol for 8 h. The washed substrate was then cleaned in an oxygen plasma oven for 60 min and screened by SERS. The cleaned plasmonic nickel foam was re-loaded with 100 µL of 25 µg/mL (6.5 × 10^−5^ M) MPN solution and allowed to stand for 15 min. The foam was then washed using deionized water, air-dried, and screened by SERS.

### 2.10. Cross-Validation of SERS Quantification of MPN with HPLC

20 µL of spiked human blood plasma (MPN concentration = 25 µg/mL (6.5 × 10^−5^ M)), were injected onto a reversed-phase C18 column (Phenomenex Kinetex 5µ XB – C18 100 Aº, size 250 × 4.60 mm) (Torrance, CA, USA). Ammonium acetate: acetonitrile (92:8, v:v) was used as a mobile phase at a flow rate of 1 mL/min for the separation of MPN. A diode array detector (DAD) was used for the detection of the separated drug. The detection wavelength was set at 298 nm [25]. For the quantification of MPN, aqueous standard solutions of the drug were prepared in a working concentration range of 0.25 to 18 µg/mL (6.5 × 10^−7^ M to 4.7 × 10^−5^ M). The samples were filtered before use. Twenty-µL aliquots of MPN standard solutions were injected onto the C18 column and screened as mentioned above. The area under the peak of the MPN band at 5.22 min was plotted against the drug concentration to develop a calibration plot.

### 2.11. Direct Detection of MPN in Spiked Blood Samples

To detect MPN in complex biological fluids, 100 µL of spiked human blood plasma sample of MPN concentration of 25 µg/mL (6.5 × 10^−5^ M), was deposited onto plasmonic nickel foam substrate and left to stand for 15 min. The substrates were washed using deionized water and air-dried before the SERS measurements where the MPN band at 1546 cm^−1^ was monitored.

### 2.12. Control Tests

Negative and positive control samples were prepared for control tests. The positive control sample was prepared by spiking MPN in blood plasma and diluting the sample with phosphate buffer saline (PBS; pH = 7.4) to the final concentration of 25 μg/mL (6.5 × 10^−5^ M). Blank (un-spiked) human blood plasma sample was diluted with PBS and used as a negative control. After, 100 µL of the positive and negative control samples were loaded onto two independent plasmonic nickel foam substrates of similar dimensions (5 × 12 mm), and allowed to stand for 15 min. The substrates were then washed with deionized water, air-dried, and screened by SERS.

### 2.13. Reproducibility of MPN Measurements by SERS

A hundred µL of 5 × 10^−4^ M (200 μg/mL) MPN solution were loaded onto a plasmonic nickel foam substrate and allowed to stand for 15 min. The substrate was then washed with deionized water, air-dried, and three independent SERS measurements were carried out by the handheld Raman spectrometer.

The reproducibility of the SERS measurements on three independent substrates was investigated by loading 100 µL of 200 μg/mL (5 × 10^−4^ M) MPN onto plasmonic nickel foam substrates of the same dimensions (5 × 12 mm) and allowing it to stand for 15 min. The substrates were washed with deionized water, air-dried, and screened by SERS.

### 2.14. SERS Detection of MPN in Blood Plasma in the Presence of Paracetamol

Paracetamol (PCM) and MPN were spiked in human blood plasma to the final concentration of 10^−6^ M of each drug. PCM was used because it is a commonly-used analgesic in many medical conditions to relieve pain. A hundred µL of spiked blood plasma sample was deposited onto plasmonic nickel foam and kept for 15 min. The substrate was then washed using deionized water and left to dry before the SERS measurement. A reference SERS spectrum of PCM was obtained by loading 100 µL of 10^−6^ M PCM (in ethanol) onto a plasmonic nickel foam. The substrate was then washed using deionized water and left to dry prior to the SERS measurement. The principal peaks were identified for both drugs and used for quantification in the complex samples.

### 2.15. Determination of MPN in Blood Plasma by HPLC-SERS

Twenty µL of spiked human blood plasma (MPN concentration = 25 μg/mL (6.5 × 10^−5^ M)) was injected onto a C18 reverse-phase column. The MPN eluate collected at 5.22 min was deposited onto a plasmonic nickel foam, allowed to stand for 15 min before air drying and screening by SERS. For the control test, 20 µL of blank human plasma (negative control) was injected and the chromatographic separation was carried out. The eluate of the negative control sample at 5.22 min at the retention time of MPN was deposited onto a plasmonic nickel foam substrate and screened by SERS.

## 3. Results and Discussion

### 3.1. Synthesis and Characterisation of Plasmonic Nickel Foam

**Figure 1:**
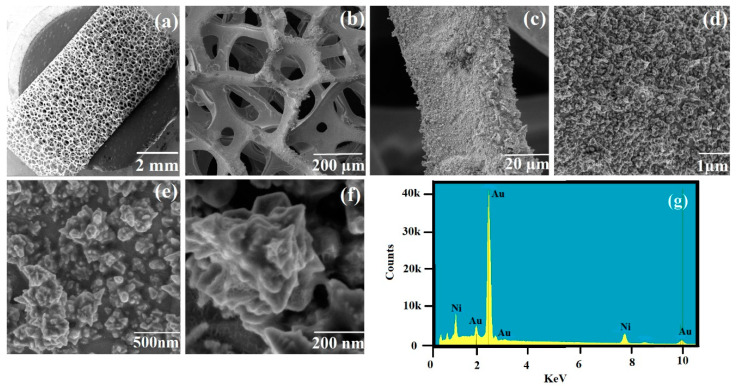
(**a**) SEM image of gold electrodeposition at 1200 s on nickel foam at 2 mm; (**b**) SEM image at 200 μm; (**c**) SEM image at 20 μm; (**d**) SEM image at 1μm; (**e**) SEM image at 500 nm; (**f**) SEM image at 200 nm; (**g**) Energy disruptive X-ray spectrum (EDX) of gold deposited nickel foam substrate.

The SEM image of the bare nickel foam shows that it has a highly porous 3D structure (Figure 1). To fabricate plasmonic nickel foam, electrochemical deposition of gold nanostructures onto the foam material was attempted at different deposition times [26]. The SEM images in Figure S1a compares the deposition of gold nanostructures at 600, 900, and 1200 s, respectively. As indicated by Figure 1, the plasmonic nickel foam showed a loading of closely packed flower-shaped gold nanostructures after electrochemical deposition of 1200 s. The high coverage of the foam allows for the efficient coupling of surface plasmons of the gold nanostructures and the formation of multiple hotspots. The strong electromagnetic field at the hotspots leads to sensitive SERS measurements [11,27]. As indicated by Figure S1b, when 2-QT used as Raman probe was screened on the plasmonic nickel foams, the highest signal intensity of the probe at 1368 cm^−1^ (CH_3_ stretch) was achieved on a plasmonic foam, which was fabricated using 1200 s electrodeposition time. The EF of the plasmonic nickel foam was measured using 2-QT as a Raman probe (Figure S1c,d) and found to be 1.6 × 10^11^. The high EF of the gold-coated nickel foam can be attributed in part to the high surface coverage of the nickel foam with closely packed gold nanostructures as indicated by the SEM images (Figure 1). The EF for SERS sensor was also calculated for the model drug MPN and found to be 6.45 × 10^4^ [27,28]. BET surface area of bare nickel foam is 5.0866 m^2^/g [29,30]. After the deposition of gold nanostructures, the surface area of the plasmonic nickel foam increased to 9.2709 m^2^/g, which allows for the coupling of numerous surface plasmons of the gold nanostructures and the formation of multiple hotspots, thus leading to high EF in the SERS measurements.

### 3.2. SERS Measurements of MPN

**Figure 2:**
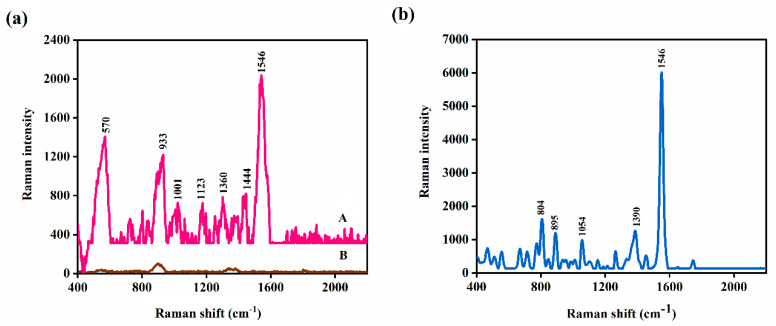
(**a**) Surface-enhanced Raman spectroscopy (SERS) spectra of (**A**) 200 μg/mL (5 × 10^−4^ M) of meropenem (MPN) on plasmonic nickel foam, and (**B**) bare plasmonic nickel foam. (**b**) Direct Raman spectrum of raw MPN.

The SERS spectrum of MPN on a plasmonic nickel foam is reported for the first time in Figure 2. The band assignments of the MPN Raman vibration modes are depicted in Table 1. The direct Raman spectrum has been given in Figure S2. All measurements were carried out in triplicates (*n* = 3).

### 3.3. SERS Quantification of MPN in Aqueous Solution and Reproducibility of the Measurements

**Figure 3:**
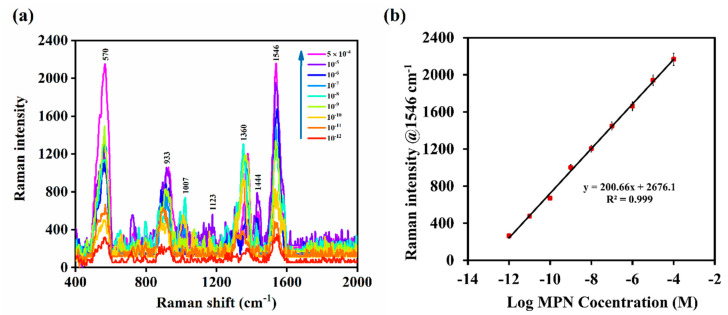
(**a**) Trend of SERS signal intensity of MPN in aqueous solution (concentration range; 5 × 10^−4^ M–1 × 10^−12^ M); (**b**) SERS calibration plot of MPN within the same concentration range.

To quantify MPN, the SERS signal intensity at 1546 cm^−1^ was monitored at different concentrations of the drug in aqueous solution and found to increase logarithmically within the concentration range of 5 × 10^−4^ M to 1 × 10^−12^ M (200 μg/mL to 3.8 pg/mL) of MPN (Figure 3a). The relationship between the signal intensity at 1546 cm^−1^ and log of the concentration of MPN was found to follow the regression equation *y* = 194.52*x* + 2642.7 (*R*^2^ = 0.997), and the limit of quantification (LOQ) was found to be 1 pM (Figure 3b). The relative standard deviation (RSD) between repeated SERS measurements in human blood plasma at 200 µg/mL (*n* = 3) was 2.86%. The RSD between SERS measurements of the MPN concentration 200 µg/mL in aqueous solution showed an RSD value of 5.5% (*n* = 3) (Figure S3). A significant reduction in the SERS signal intensity at 1546 cm^−1^ was observed when the measurements were repeated on the same substrate over 24 h (Figure S4). This is attributed to the low stability and rapid degradation of the drug [21,32]. Therefore, the SERS method can be utilized to monitor the concentration of MPN formulations within the pharmaceutical industry and points of care.

### 3.4. Direct SERS Quantification of MPN in Human Plasma

To demonstrate the potential of the new plasmonic nickel foam for the TDM, it was used for the direct SERS screening of MPN (25 µg/mL) in the spiked human blood plasma sample. As indicated by Figure S5, the diagnostic Raman bands of MPN were observed in the SERS spectra of the spiked samples and was absent in blank (un-spiked) human blood plasma. For the quantification of MPN in blood, a standard plot was constructed within the MPN concentration range of 5 × 10^−4^ M to 10^−12^ M (200 μg/mL to 3.8 pg/mL). The calibration plot followed the linear regression equation *y* = 260.83*x* + 3579.8 (*R*^2^ = 0.997) and LOQ was 1 pM (Figure S6) [5].

**Figure 4:**
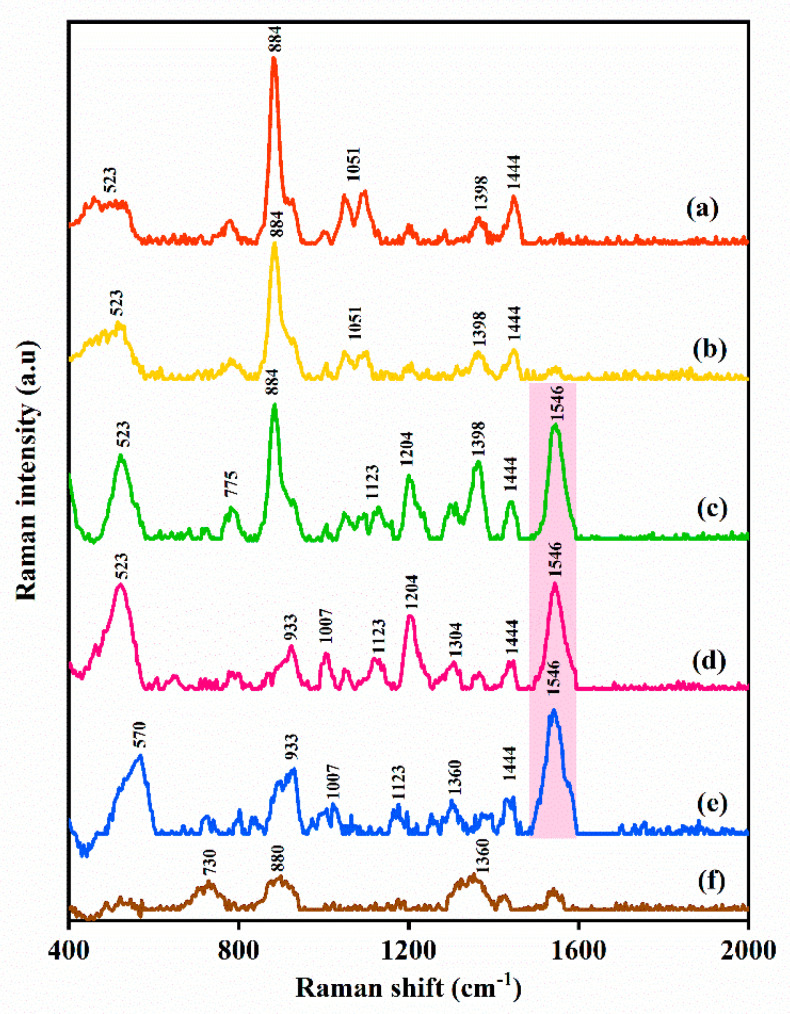
SERS spectra of (**a**) human blood plasma spiked with paracetamol (PCM); (**b**) PCM reference spectrum in ethanol; (**c**) human blood plasma spiked with MPN and PCM; (**d**) human blood plasma spiked with MPN (positive control); (**e**) reference MPN spectrum in aqueous solution; (**f**) blank human plasma (negative control).

The SERS method was also utilized for the determination of MPN in the presence of other drug (PCM) in human blood plasma. MPN and PCM (25 µg/mL each) were spiked into a blood plasma sample and an aliquot of the sample was loaded onto a plasmonic nickel foam and screened by SERS (*n* = 3) (Figure 4). As shown by Figure 4c, the diagnostic Raman bands of PCM and MPN at 884 cm^−1^ and 1546 cm^−1^, respectively were easily distinguishable in the acquired SERS spectrum of the sample. MPN was quantified and found to be 24.4 μg/mL and the percentage error between the measured and the certified concentration in the sample was 2.4%. Therefore, the SERS determination of MPN in the sample was not affected by the presence of PCM.

### 3.5. Cross Validation with HPLC-DAD

For cross validation of the SERS measurements, an HPLC-DAD method was used for the determination of MPN [25]. As indicated by Figure S7, the retention time of MPN was found to be 5.22 min. A calibration plot was established within the MPN concentration range of 0.25 µg/mL to 18 µg/mL (Figure S7) [33]. The correlation between the area under the peak at 5.22 min and the concentration of the MPN followed the linear regression equation *y* = 1200.66*x* + 2676.1 (*R*^2^ = 0.999) and LOQ was 0.25 µg/mL [34]. The chromatographic method was used to screen MPN in a spiked plasma sample. The concentration of MPN in the sample was found to be 24.6 µg/mL by HPLC-DAD. Another aliquot was screened by SERS and the MPN concentration was found to be 24.5 µg/mL. Therefore, the % agreement between the two methods was 98%, which indicates that the two methods are comparable.

### 3.6. Determination of MPN by HPLC-SERS

**Figure 5:**
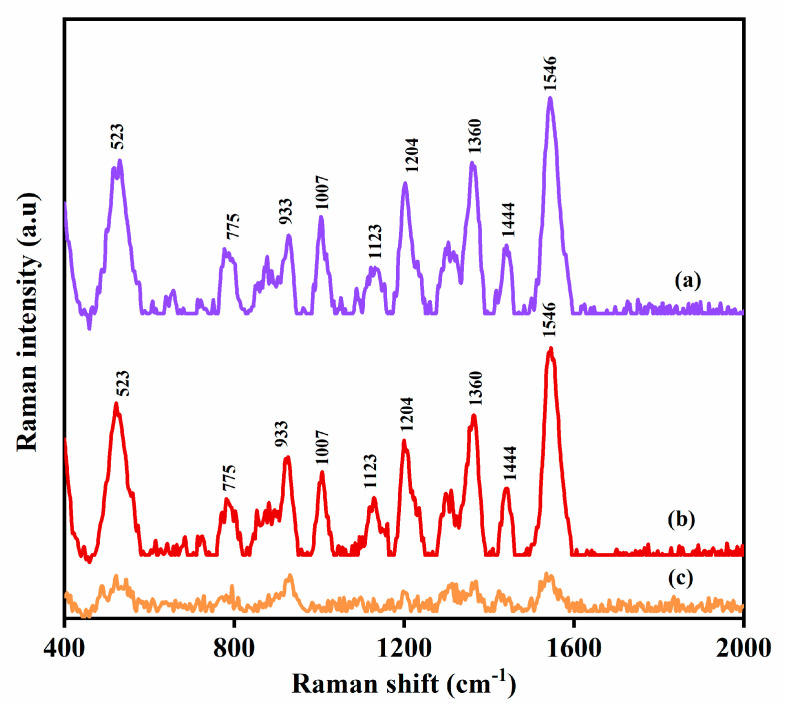
SERS spectra of: (**a**) MPN in human blood plasma; (**b**) SERS spectrum after separation by HPLC and detection by SERS in an HPLC-SERS arrangement; (**c**) blank human blood plasma.

SERS can provide molecular structure identification of analytes. Therefore, the plasmonic nickel foam was utilized as a sensor to replace the diode array detector in an HPLC-SERS arrangement to provide a molecular structure identification of the target analyte similar to MS detector [2]. The HPLC-SERS method was used to determine MPN (25 µg/mL) in a spiked human blood plasma sample. The chromatographic separation was carried out and the eluate fraction at 5.22 min was directed onto the plasmonic nickel foam and screened by SERS (Figure 5). As implied by Figure 5b, the SERS spectrum of MPN was obtained after the chromatographic separation of the spiked human plasma sample and the concentration was quantified to be 24.6 µg/mL. Therefore, the % agreement between the certified concentration and that quantified by HPLC-SERS was 98.4%.

### 3.7. Recycling of SERS Substrate

To reduce the cost of the SERS measurements, the used plasmonic nickel foam was recycled by washing it with absolute ethanol and cleaning in an oxygen plasma oven. As indicated by Figure S8, no SERS spectrum was detected from the recycled plasmonic nickel foam. This result revealed that the MPN analyte was efficiently removed from the substrate. The recycled substrate was reloaded with a fresh aliquot of MPN (concentration 200 µg/mL (5 × 10^−4^ M)) and re-screened by SERS. As indicated by Figure S8, the SERS spectrum of MPN was detected on the recycled substrate. The concentration of MPN was quantified by SERS and found to be 24.5 µg/mL. This result indicated that the recycling process did not compromise the SERS performance of the substrate.

To determine the error introduced by the automatic background correction of the Raman spectrometer, gold-coated nickel foam was loaded with MPN (5 × 10^−4^ M) and the SERS measurements carried out by the handheld device. The Raman signal intensity at 1546 cm^−1^ was determined from corrected and un-corrected spectra (Figure S9), and the concentration of MPN was calculated using the linear regression equation in Section 3.3. The percent error between the concentration calculated from the uncorrected and corrected spectra was found to be 0.23%.

## 4. Conclusions

This study has demonstrated the application of a new plasmonic nickel foam substrate for the SERS detection of drugs in biological matrices. A simple electrodeposition method was used to deposit a flower-shaped forest of gold nanostructures on nickel foam. The new SERS substrate showed a high enhancement factor of 1.6 × 10^11^ M. The substrate was used for direct SERS quantification of MPN in human blood plasma (LOQ = 1 pM) and showed high sensitivity. The substrate was also coupled to HPLC in an HPLC-SERS arrangement to replace the DAD detector and give molecular structure identification of the analyte. The new SERS substrate demonstrated good reproducibility (RSD = 2.86%) in human blood plasma and (RSD = 5.5%) in aqueous solution at 200 µg/mL. The cost of MPN analysis was reduced by recycling the used substrate by a simple method. Hence, the new SERS substrate has robust potential for rapid screening, molecular structure identification, and therapeutic monitoring of drugs after their separation in biological fluids.

## Figures and Tables

**Table 1:** Band assignment of the SERS modes of MPN.

Raman Shift (cm^−1^)	Band Assignment	Reference
1546	CH^3^ in dimethylcarbamoyl group	[31]
1444	C-N in dimethylcarbamoyl group	[31]
1360	CH in hydroxyethyl group, CH in pyrrolidine ring	[31]
1123	CH twist, C-N stretch in pyrrolidine ring	[31]
1007	C-C in β-lactam ring, CH in trans-hydroxyethyl group	[31]
933	-CO symmetric stretch C-C between β-lactam and dimethylcarbamoyl group CN in β-lactam	[5]
570	O-H b in carboxylic group	[31]

## Supplementary Materials

The following are available online at https://www.mdpi.com/2079-4991/10/9/1756/s1, Figure S1. (a) SEM images of gold deposition on nickel foam at different acquisition times 600, 900, and 1200 s (resolution 1 μm); (b) SERS spectrum of 2-QT (10^−6^ M) at different acquisition times 600, 900, and 1200 s; (c) 2-QT on bare nickel foam substrate (10^−2^ M); (d) 2-QT on plasmonic nickel foam substrate (10^−12^ M). Figure S2. Chemical structure of: (a) meropenem; (b) paracetamol. Figure S3. (a) Reproducibility of SERS measurements in human blood plasma (RSD = 2.86%); (b) reproducibility of signals on SERS measurements in aqueous solution (RSD = 5.52%) (MPN conc. 200 µg/mL, 5 × 10^−4^ M). Figure S4. Degradation of drug in aqueous medium over time measured by plasmonic nickel SERS sensor. Figure S5. SERS spectra of (a) meropenem (MPN) spiked in human blood plasma; (b) MPN in aqueous solution; (c) blank plasmonic nickel substrate. Figure S6. (a) Trend of SERS signal intensity of MPN in human blood plasma in concentration range of 5 × 10^−4^ M to 1 × 10^−12^ M; (b) SERS calibration plot of MPN in human blood plasma within the same concentration range. Figure S7. (a) MPN high-performance liquid chromatography (HPLC) chromatograms (*n* = 3) (RT = 5.22 min): (i) blank solvent, (ii) standard MPN in aqueous solution, (iii) blank human plasma, (iv) MPN spiked in human plasma; (b) calibration curve of MPN by HPLC (working concentration range 0.25 to 18 µg/mL). Figure S8. SERS spectra of (a) (i) MPN on plasmonic nickel SERS substrate (ii) SERS substrate after desorption of MPN (iii) Fresh aliquot of MPN on recycled SERS substrate (b) Repeated cycles of measurements. Figure S9. SERS spectra of 5 × 10^−4^ M MPN on plasmonic nickel foam. The spectra were acquired by the handheld Raman spectrometer (a) using automatic background correction, (b) without background correction.
