# Supplementary material for: Gold-Deposited Nickel Foam as Recyclable Plasmonic Sensor for Therapeutic Drug Monitoring in Blood by Surface-Enhanced Raman Spectroscopy
Source: Nanomaterials (Basel). 2020 Sep 6;10(9):1756. doi: 10.3390/nano10091756 (PMC7558188; doi:10.3390/nano10091756)
Supplement: Supplementary file 1 [file nanomaterials-10-01756-s001.pdf]

nanomaterials

Supplementary Materials

# Gold-Deposited Nickel Foam as Recyclable Plasmonic Sensor for Therapeutic Drug Monitoring in Blood by Surface-Enhanced Raman Spectroscopy

**Saiqa Muneer [1], Daniel K. Sarfo [1], Godwin A. Ayoko [1], Nazrul Islam [2] and Emad L. Izake [1,*]**

[1] School of Chemistry and Physics, Science and Engineering Faculty, Queensland University of Technology, 2 George St, Brisbane, QLD, 4000, Australia; saiqa.muneer@hdr.qut.edu.au (S.M.); daniel.sarfo@qut.edu.au (D.K.S.); g.ayoko@qut.edu.au (G.A.A.)

[2] School of Clinical Sciences, Faculty of Health, Queensland University of Technology, 2 George St, Brisbane, QLD, 4000, Australia; nazrul.islam@qut.edu.au

* Correspondence: e.kiriakous@qut.edu.au; Tel.: +61-7-3138-2501

---

## Surface-Enhanced Raman spectroscopy (SERS) Quantification Using Raman Probe

SERS enhancement factor was calculated by the deposition of $10^{-12}$ M solution of 2-Quinoline thiol, 2-QT (Raman probe) on plasmonic nickel substrate and $10^{-2}$ M on bare Ni-F for 15 min. The acquired SERS spectra of QT before and after deposition of gold on SERS substrate are depicted in Figure S1.

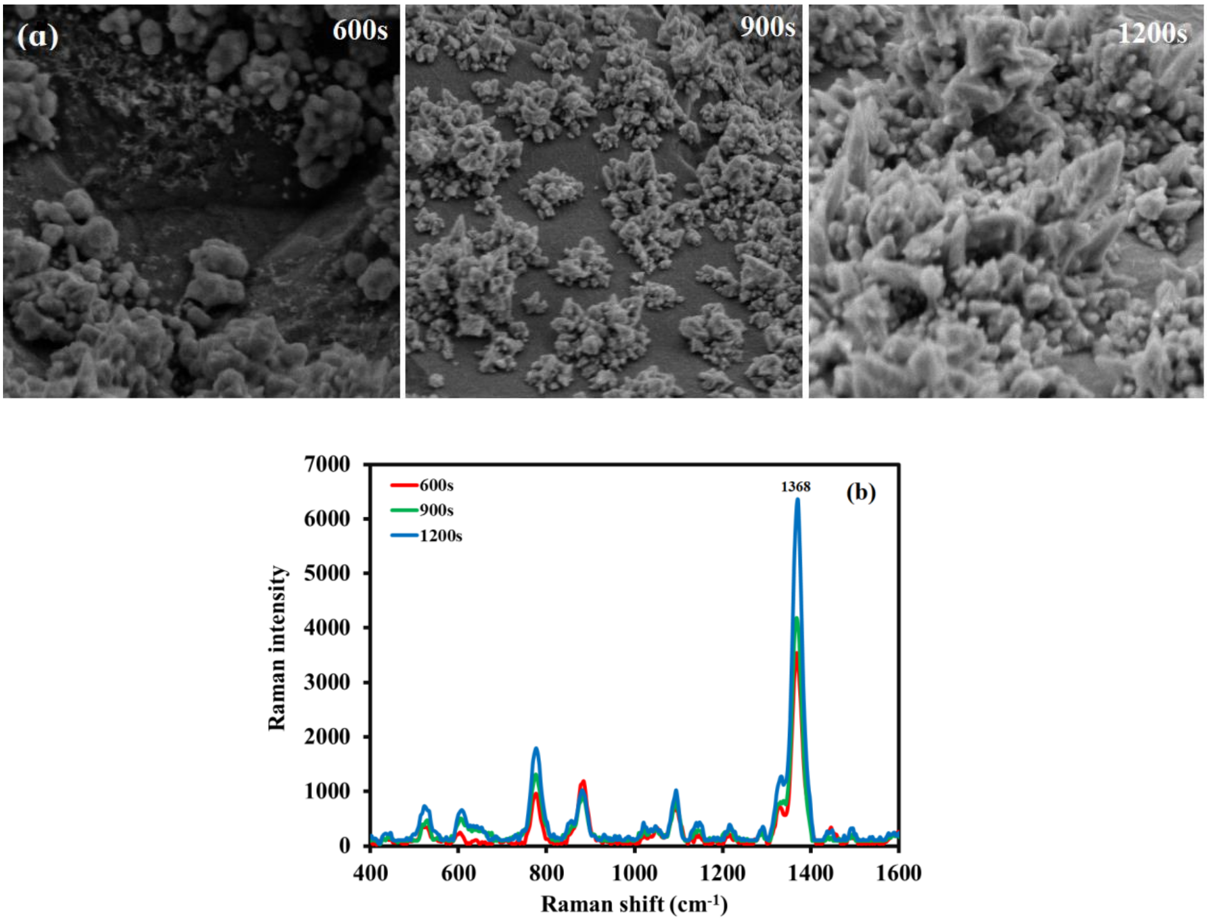

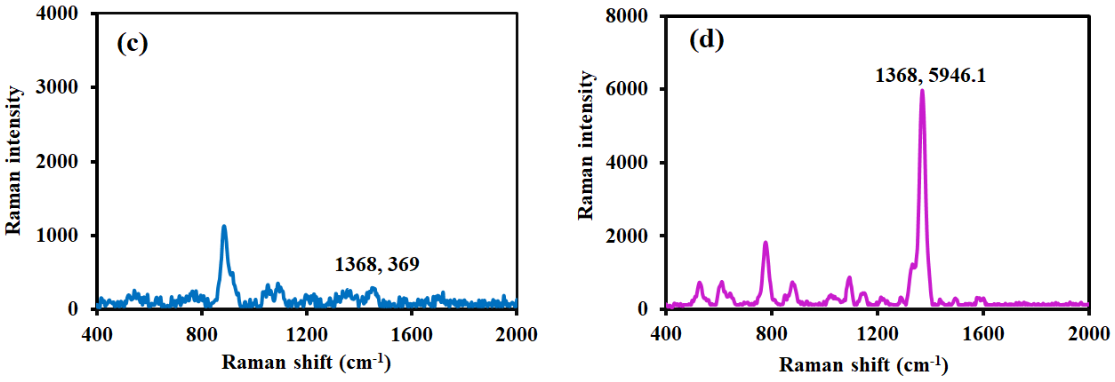


**Figure S1.** (**a**) SEM images of gold deposition on nickel foam at different acquisition times 600, 900, and 1200 s (resolution 1 µm); (**b**) SERS spectrum of 2-QT ($10^{-6}$ M) at different acquisition times 600, 900, and 1200 s; (**c**) 2-QT on bare nickel foam substrate ($10^{-2}$ M); (**d**) 2-QT on plasmonic nickel foam substrate ($10^{-12}$ M).

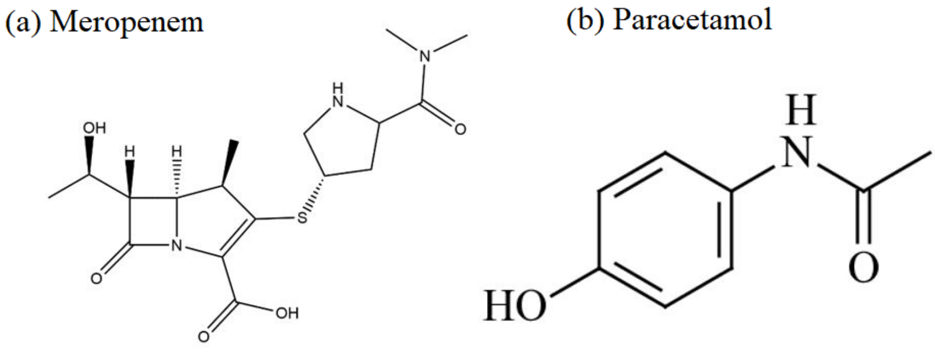


**Figure S2.** Chemical structure of: (**a**) meropenem; (**b**) paracetamol.

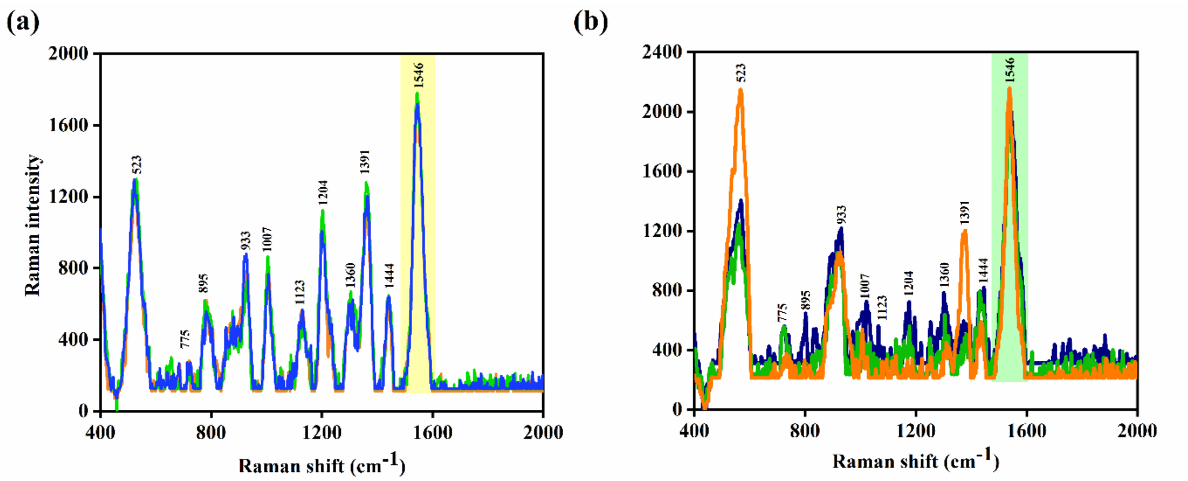


**Figure S3.** (**a**) Reproducibility of SERS measurements in human blood plasma (RSD = 2.86%); (**b**) reproducibility of signals on SERS measurements in aqueous solution (RSD = 5.52%) (MPN conc. 200 µg/mL, 5 × $10^{-4}$ M).

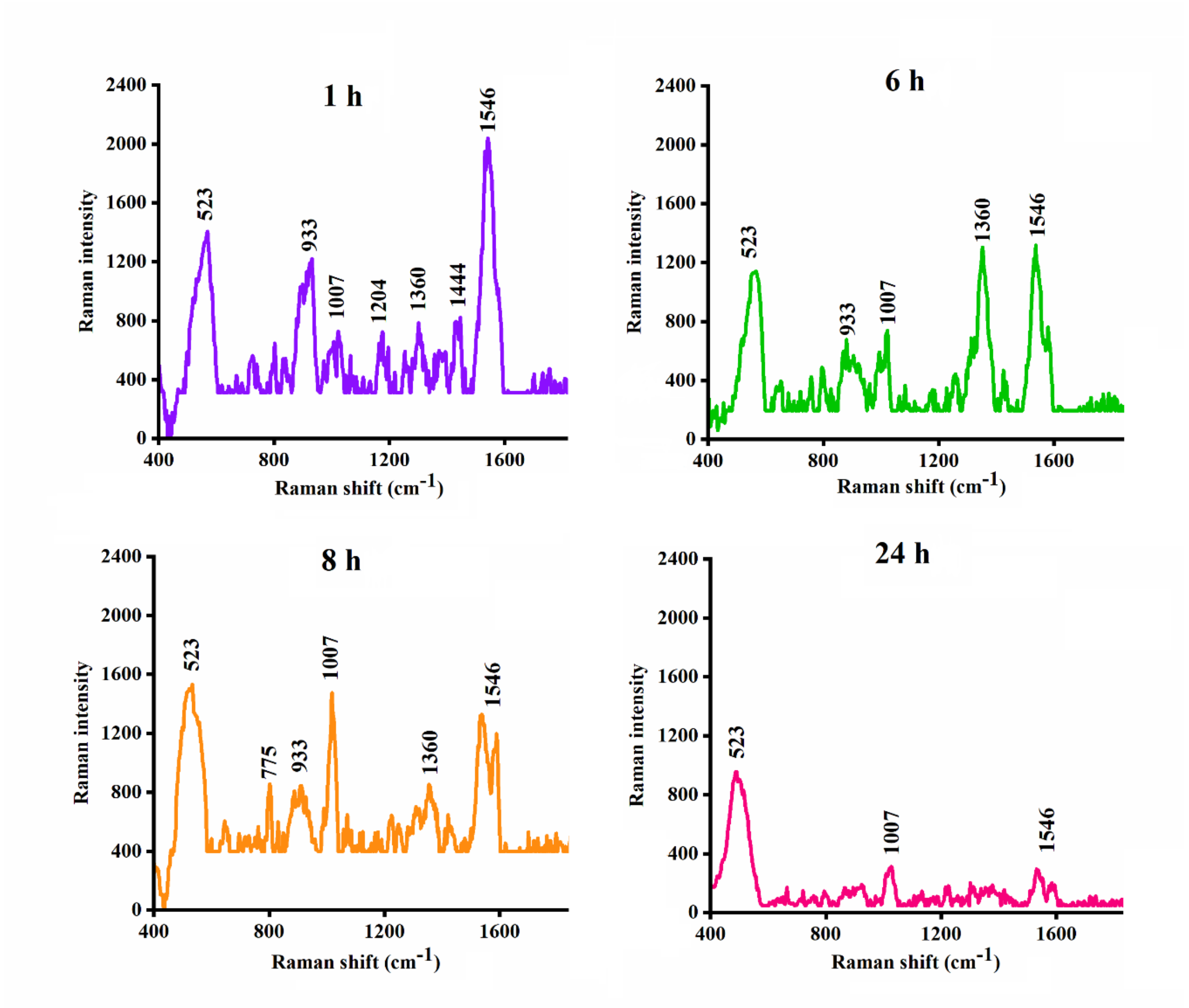


**Figure S4.** Degradation of drug in aqueous medium over time measured by plasmonic nickel SERS sensor.

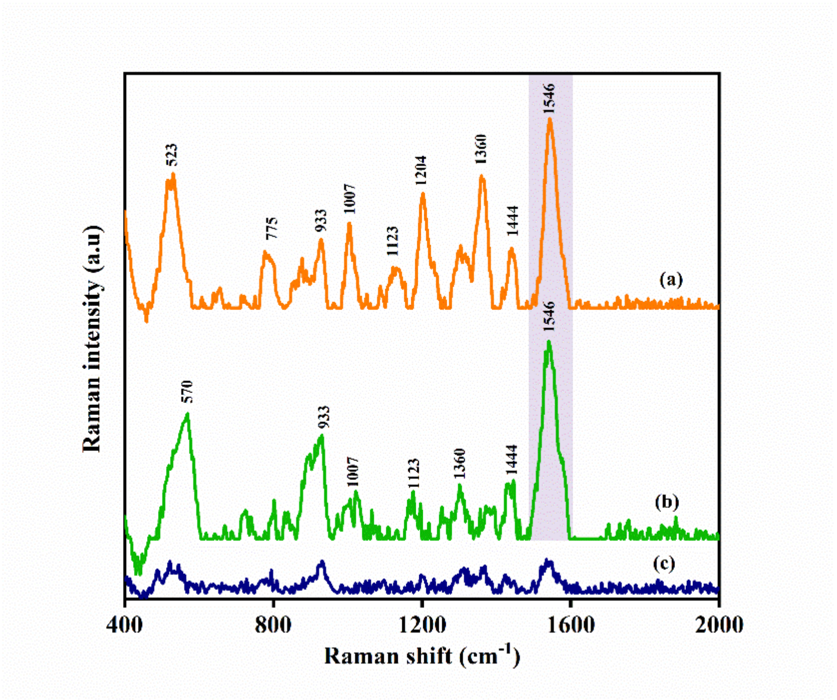

**Figure S5.** SERS spectra of (**a**) meropenem (MPN) spiked in human blood plasma; (**b**) MPN in aqueous solution; (**c**) blank plasmonic nickel substrate.

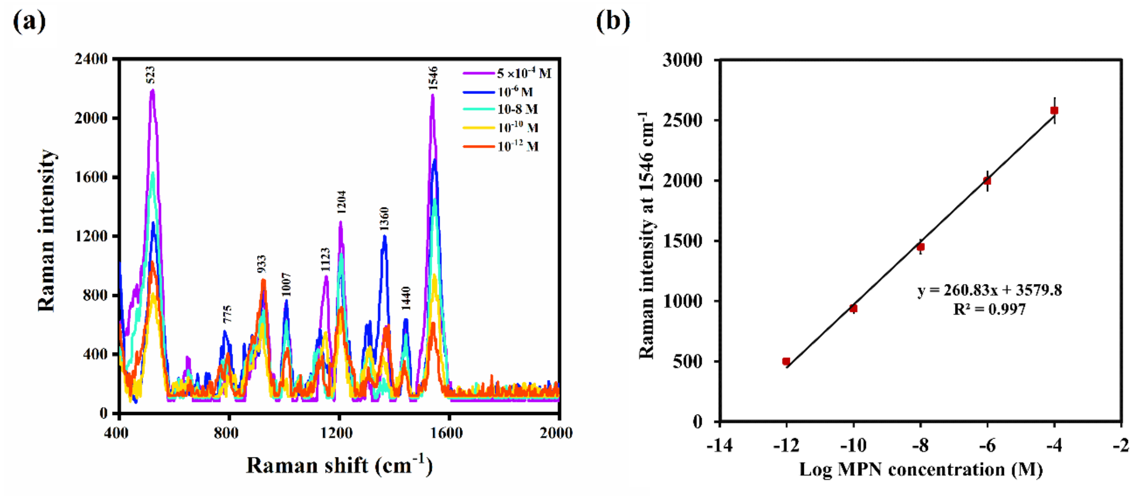


**Figure S6.** (**a**) Trend of SERS signal intensity of MPN in human blood plasma in concentration range of 5 × $10^{-4}$ M to 1 × $10^{-12}$ M; (**b**) SERS calibration plot of MPN in human blood plasma within the same concentration range.

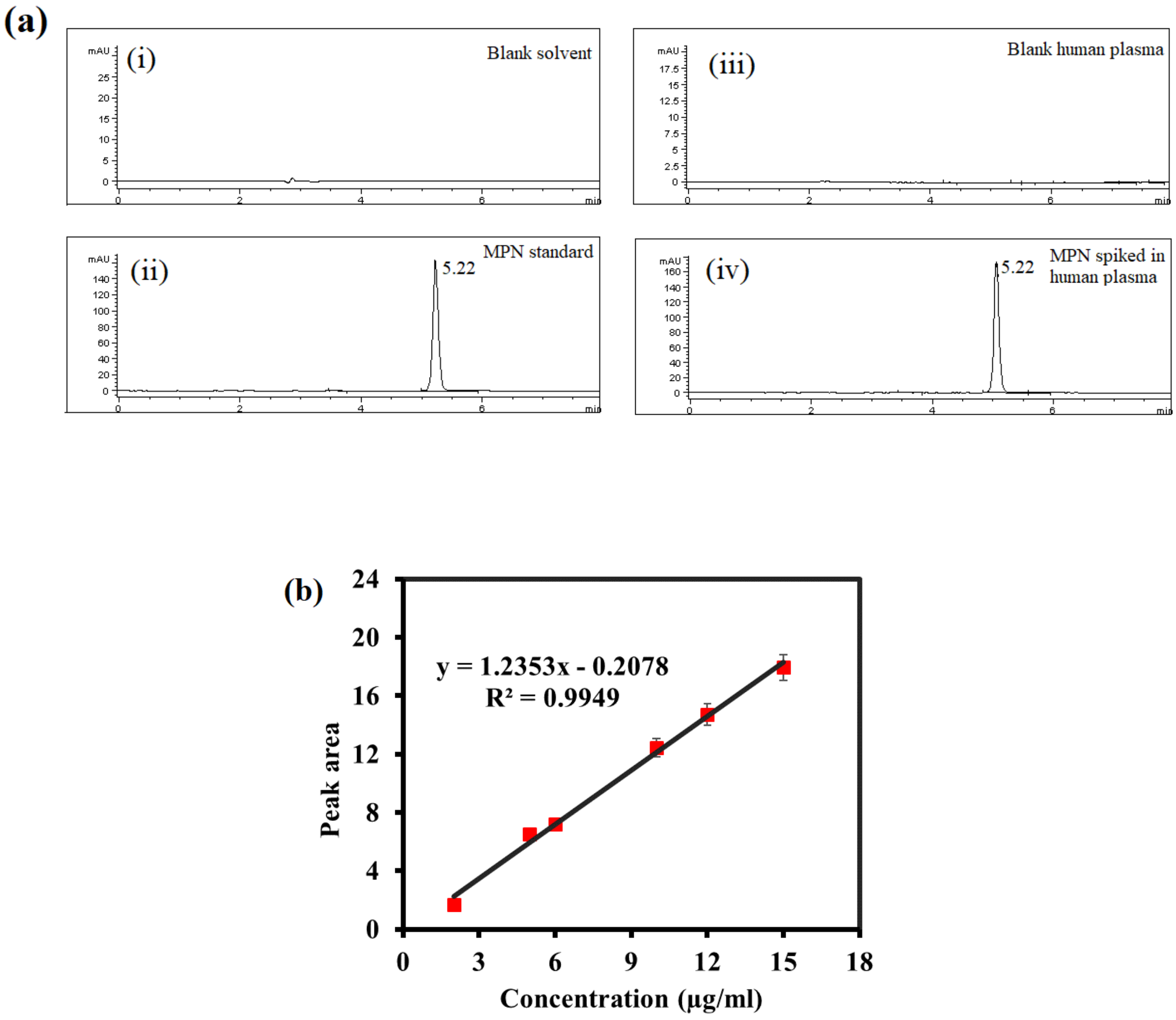

**Figure S7.** (**a**) MPN high-performance liquid chromatography (HPLC) chromatograms (*n* = 3) (RT = 5.22 min): (i) blank solvent, (ii) standard MPN in aqueous solution, (iii) blank human plasma, (iv) MPN spiked in human plasma; (**b**) calibration curve of MPN by HPLC (working concentration range 0.25 to 18 μg/mL).

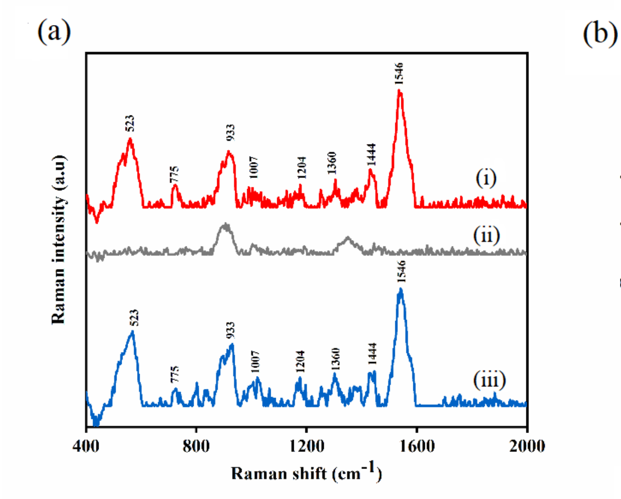


**Figure S8.** SERS spectra of (**a**) (i) MPN on plasmonic nickel SERS substrate (ii) SERS substrate after desorption of MPN (iii) Fresh aliquot of MPN on recycled SERS substrate (**b**) Repeated cycles of measurements.

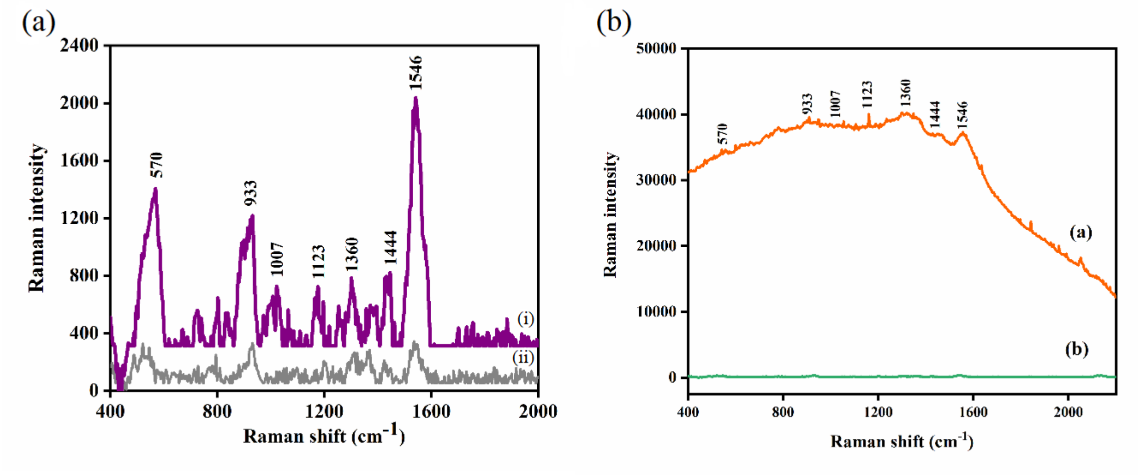


**Figure S9.** SERS spectra of 5 × $10^{-4}$ M MPN on plasmonic nickel foam. The spectra were acquired by the handheld Raman spectrometer (**a**) using automatic background correction, (**b**) without background correction.

## Calculation of Limit of Quantification (LOQ) by SERS

For SERS measurements, limit of quantification (LOQ) is calculated by the formula; LOQ = 10* (standard deviation of low concentration/slop of calibration curve) [1].

## References

[1] Shrivastava, A.; Gupta, V.B. Methods for the determination of limit of detection and limit of quantitation of the analytical methods. *Chron. Young. Sci.* **2011**, *2*, 21–25.
